# The Sleep of Schoolboys

**Published:** 1905-12-30

**Authors:** 


### The Sleep of Schoolboys.

A letter has recently appeared in the *Times*, signed by several representative members of the profession, and expressing the belief of the signatories that the hours of sleep at schools, especially for boys under sixteen years of age, are ordinarily insufficient for the proper growth and development of their brain tissue, and indicating, not obscurely, that very widespread mischief is thus produced. A letter of such a kind, in the terms of which many persons are called upon to agree, is of necessity somewhat general in its expressions; but the writers do not in the least disguise their opinion of the gravity of the conditions to which they direct attention. They declare that the harm done by giving too little sleep to growing boys is not only that it lessens their power of fixing their attention on work, and causes slackness and weariness in games, but also that it is a definite factor in the causation of intellectual inefficiency, which may be far-reaching in its effects. They point out that a minimum of nine hours of unbroken rest in summer and nine and a half in winter is held by those who have most closely studied the subject to be needed by the average boy of from thirteen to sixteen. They say that it is not sufficient to send the younger boys early to bed in dormitories in which the older boys cause a disturbance by coming up later; and that a definite wrong is inflicted on growing boys by giving them only the amount of sleep that is needed by those who have reached maturity. They declare that, if this be done, it is almost certain that the majority of boys will leave school less well equipped for the struggle of life, both in body and mind, than might have been the case if more generous hours of rest had been accorded to them during their years of development. Finally, they assert that the question is one which should interest all parents who have a boy at school, as well as all schoolmasters, and express their hope that a much-needed reform in this essential part of school hygiene will not be long postponed.

Attention is also called in the letter to a paper (by Dr. T. D. Acland) read at the last annual meeting of the Association of Medical Officers of Schools, in which the question at issue is much more fully and completely dealt with than the limits of a letter would allow; and which has been sent by the Council of the Association to the headmaster and the chairman of the governing body of all the principal schools in the country. In this paper Dr. Acland shows that insufficient sleeping time is almost the rule in English schools, which in this respect offer a marked contrast to those of America; and he quotes a very significant statement from Dr. Welldon, formerly headmaster of Harrow, with reference to "the number of clever, brilliant boys he had known, who, in after life, had absolutely disappeared." He further quotes the observation of Dr. Dukes upon this statement, to the effect that Dr. Welldon "did not recognise that the immature brain tissue of these boys had been exhausted before they attained manhood." Dr. Dukes clearly attaches its full meaning to the declaration of the letter that want of sufficient sleep is a definite factor in the causation of intellectual inefficiency which may be far-reaching in its effects; and this statement, especially if we may be permitted to read between its lines, is clearly one of the highest possible importance. Dr. Dukes has more than once called attention to the fact that the education of the upper and middle classes of Great Britain is the only pursuit for which those who are to follow it receive no training and frequently possess no competence; and it can hardly be doubted that an intellectual inefficiency of these classes, brought about by absence of physiological knowledge on the part of their preceptors, is a serious national evil. We have not much hope of a reform in schools, unless it be absolutely insisted upon by parents, upon whom we would strongly urge the necessity of being guided by knowledge, and of disregarding the outcries of pedantic ignorance.

